# The Toll Route to Structural Brain Plasticity

**DOI:** 10.3389/fphys.2021.679766

**Published:** 2021-07-05

**Authors:** Guiyi Li, Alicia Hidalgo

**Affiliations:** Plasticity and Regeneration Lab, School of Biosciences, University of Birmingham, Birmingham, United Kingdom

**Keywords:** *Drosophila*, structural brain plasticity, neurodegeneration, adult neurogenesis, neurotrophin, Toll, TLR, homeostasis

## Abstract

The human brain can change throughout life as we learn, adapt and age. A balance between structural brain plasticity and homeostasis characterizes the healthy brain, and the breakdown of this balance accompanies brain tumors, psychiatric disorders, and neurodegenerative diseases. However, the link between circuit modifications, brain function, and behavior remains unclear. Importantly, the underlying molecular mechanisms are starting to be uncovered. The fruit-fly *Drosophila* is a very powerful model organism to discover molecular mechanisms and test them *in vivo*. There is abundant evidence that the *Drosophila* brain is plastic, and here we travel from the pioneering discoveries to recent findings and progress on molecular mechanisms. We pause on the recent discovery that, in the *Drosophila* central nervous system, Toll receptors—which bind neurotrophin ligands—regulate structural plasticity during development and in the adult brain. Through their topographic distribution across distinct brain modules and their ability to switch between alternative signaling outcomes, Tolls can enable the brain to translate experience into structural change. Intriguing similarities between Toll and mammalian Toll-like receptor function could reveal a further involvement in structural plasticity, degeneration, and disease in the human brain.

## Introduction

The brain can change throughout life. Cells, neurites (axons and dendrites), and synapses are generated with experience and learning as we adapt to the environment. They are also eliminated, maintaining neural circuit stability and normal behavior. The balance between the former, known as structural plasticity, and the latter, known as structural homeostasis, characterizes brain health, and its breakdown accompanies a brain disease—such as brain tumors, psychiatric disorders, and neurodegenerative diseases (e.g., Alzheimer’s and Parkinson’s diseases). Structural plasticity and homeostasis may reveal how the brain works, as brain function relies on structural changes to cells.

Structural plasticity is often understood to be “hebbian,” whereby co-active synapses are stabilized, reinforcing connections. Structural homeostasis is also known as “non-hebbian plasticity” and includes elimination and “compensatory plasticity,” such as increases in synapse number as arborizations decrease, to deliver normal function. Synapses can also manifest physiological plasticity such as synaptic potentiation, depression, or homeostatic plasticity (e.g., involving regulatory adjustments in neurotransmitter release or post-synaptic excitability to maintain a normal function). Here we will deal only with structural changes.

Mammalian cortical plasticity has long been known (Feldman and Brecht, 2005). Classical experiments by Wiesel and Hubel showed that synapses and neurites are modified in response to neuronal activity and experience (Wiesel and Hubel, 1965). When kittens were deprived of light in one eye, arborizations from active neurons originating from the open eye outcompeted the silent ones from the closed eye, altering the pattern of innervation in the cortex. Importantly, recurrent cycles of light deprivation and exposure did not cause spine elimination to restore original spine profiles but instead maintained and built on newly formed synapses (Hofer et al., 2009). This suggests that structural changes in neurons may store information from the past, enabling the brain to adapt and respond in the future. However, plasticity may not necessarily be adaptive nor beneficial, but just a consequence of available connectivity opportunities for neurons (Feldman and Brecht, 2005).

Structural plasticity can also involve neurogenesis. Enriched environments, exercise, voluntary running, and learning can induce neurogenesis in the adult mammalian brain (Gage, 2004, 2019; Deng et al., 2010). In mammals, neurogenesis occurs in restricted brain areas, including the dentate gyrus of the hippocampus and the subventricular zone generating olfactory bulb neurons (Eriksson et al., 1998; Spalding et al., 2013; Goncalves et al., 2016; Simoes and Rhiner, 2017). Hippocampal neurogenesis may be required for spatial navigation and learning and memory to encode time into memory and prevent interference with old memories (Deng et al., 2010). However, this remains to be established.

The molecular mechanisms underlying structural brain plasticity and homeostasis remain virtually unknown. The neurotrophin BDNF and its receptor TrkB are currently the key factors known to be involved, and there is evidence that Toll-like receptors (TLRs) could also be involved (Lu et al., 2005, 2013; Okun et al., 2011; Park and Poo, 2013). Progress in understanding the link between molecular mechanisms, circuits, structural brain plasticity, homeostasis, and brain health in mammals has long been challenging.

The fruit-fly *Drosophila* is a powerful model organism to discover molecular mechanisms and link genes to neurons, circuits, and behavior. Here we review pioneering work on structural brain plasticity and homeostasis in *Drosophila*, current evidence of underlying molecular mechanisms, and how they could relate to the mammalian brain.

## Structural Plasticity and Homeostasis in the Adult *Drosophila* Brain

### Structural Plasticity: Brain Size Is Variable and Regulated by Experience

Pioneering work by Martin Heisenberg’s lab started the structural plasticity journey through the *Drosophila* brain. Gerhard Technau showed that, in *Drosophila*, fiber number in the adult mushroom body peduncle can increase by 15% at 1 week after adult fly eclosion, and this was influenced by environmental stimuli (Technau, 1984). Flies reared in isolation, constant darkness, or olfactory deprivation had a significantly reduced fiber number (Technau, 1984). In fact, Kenyon cell number in wild-type flies is variable and can change depending on larval growth conditions and experience in the first week of adult fly life (Balling et al., 1987; Heisenberg et al., 1995). Brain size also changed with experience. Eclosed adult flies were kept either in isolation or single sex populations (i.e., deprived environment) or in large population cages or mixed sexes (i.e., enriched) for 19 days, and then the brain volume was measured. The volume of various brain structures increased in enriched vs. deprived flies, including the mushroom bodies, calyces, and the visual system lamina, medulla, and lobula (Heisenberg et al., 1995). Furthermore, compared to flies reared in constant darkness, stimulation of the visual system by breeding flies in constant light during a critical period spanning the first 5 days after eclosion increased the volume of the lamina and lobula plate, the size of photoreceptors and glial cells, the mushroom body calyces, and the central complex (Barth and Heisenberg, 1997; Barth et al., 1997). These pioneering studies did not resolve the cellular bases of structural brain plasticity. Some limited proliferation was detected in adult brains, but it was deemed to be insufficient to explain the extent of structural brain change (Technau, 2007). This work altogether showed that brain size can be modified by experience and that different experiences can affect brain regions differentially.

Findings from the olfactory system provided independent evidence for structural plasticity during the critical period in other brain regions (Devaud et al., 2003). The olfactory glomeruli continue to grow between days 1 and 12 of adult fly life, and the volume of particular glomeruli was influenced by experience—i.e., exposure to odorant—concomitantly with the maturation of odorant-induced behavior (Devaud et al., 2003). Similarly, when flies were exposed to a high concentration of carbon dioxide (CO_2_) during the critical period, the volume of the CO_2_-specific V glomerulus in the antennal lobe significantly increased (Sachse et al., 2007). This structural change was reversible after recovering at normal air exposure for another 5 days. By contrast, CO_2_ exposure after the critical period did not cause a significant structural alteration in the V glomerulus (Sachse et al., 2007). The microglomeruli that form as projection neurons from the antennal lobe connecting to the Kenyon cell dendrites at the calyx are also modified by experience. After conditioning young flies with an odorant (11-*cis*-vaccenylacetate) to form long-term memory, the size of the microglomeruli responding to this odorant decreased, but the number of such microglomeruli increased (Baltruschat et al., 2021). This demonstrated that new synaptic boutons are formed during learning and for long-term memory and that structural change is linked to brain function.

**FIGURE 1:**
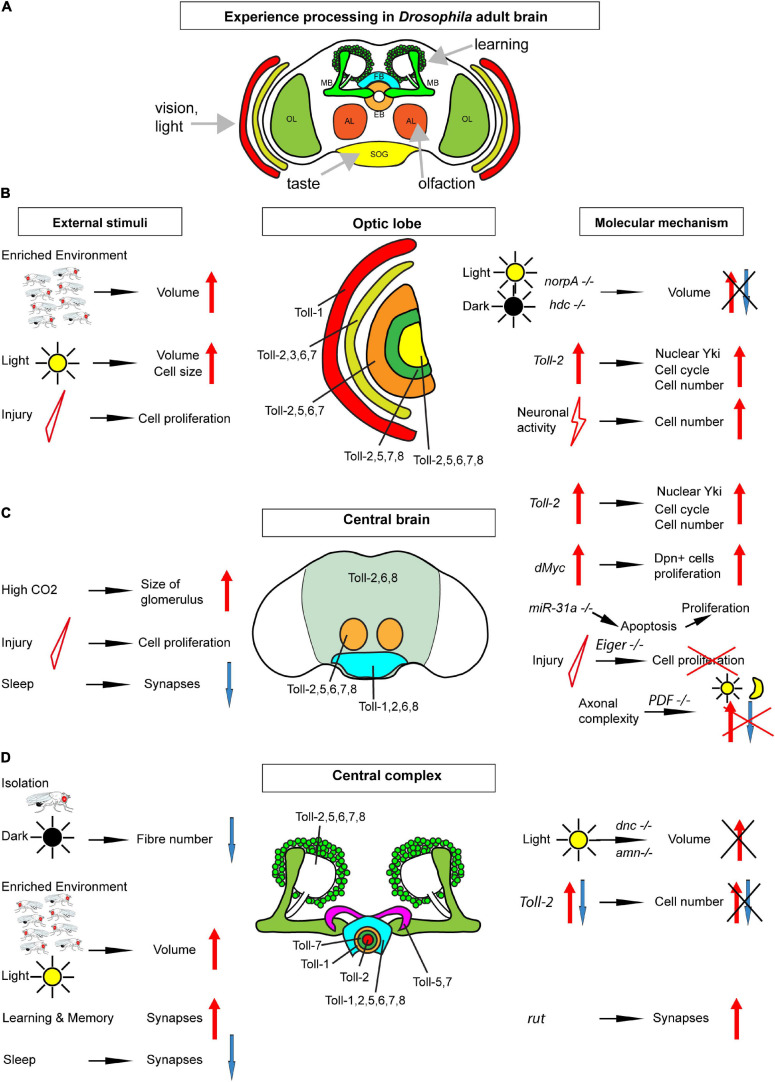
Map of Toll receptors in a *Drosophila* adult brain. **(A)** *Drosophila* adult brain illustrating some modules receiving sensory input and involved in learning. **(B)** Optic lobe (including retina, lamina, medulla, lobula, and lobula plate from outer to inner layers). **(C)** Central brain [including neuropiles (pale green), antennal lobes (orange), and sub-esophageal ganglion (bright blue)]. **(D)** Central complex: mushroom bodies (green) ellipsoid body in rings, fan-shaped body (bright blue), and protocerebral bridge (purple). Each brain module expresses different Tolls or a combination of Tolls; thus, they could be regulated differentially in distinct brain modules. From **(B–D)**, the key findings involved in structural brain plasticity are summarized with external stimuli on the left and molecular manipulations and mechanisms on the right. OL, optic lobe; MB, mushroom body; FB, fan-shaped body; EB, ellipsoid body; AL, antennal lobe; SOG, sub-esophageal ganglion.

To conclude, multiple regions within the adult brain—visual and olfactory systems and central complex—can be modified by experience (Figure 1). Whether this is restricted to a critical period within the first week of adult fly life or continues throughout the life course remains to be tested further and established. Most studies show that plasticity takes place within a critical period in the first week of adult life but do not carry out test beyond. Heisenberg showed that, at least under some conditions, the brain remains plastic beyond this period and into at least the second week (Heisenberg et al., 1995). This varies across brain regions, suggesting that different mechanisms with distinct dynamics regulate plasticity in the different brain domains (Heisenberg et al., 1995).

### Structural Homeostasis: Elimination and Compensation

Structural homeostasis was initially reported in the adult brain of *Musca domestica* (Kral and Meinertzhagen, 1989). Eclosed adult flies were subjected for 2 days to two regimens: they were either kept in constant darkness or stimulated by flickering green light (Kral and Meinertzhagen, 1989). As a result, the synapse number in lamina neurons that connect to photoreceptors decreased with light exposure and increased in flies reared in the dark (Kral and Meinertzhagen, 1989). Thus, neuronal stimulation provoked synapse elimination, and the lack of stimulation increased synapse formation. Subjecting adult flies to cold-shock also decreased the synapse number, and synapses could be recovered by transferring the flies to warmer temperatures (Brandstatter and Meinertzhagen, 1995). These findings provided evidence for structural homeostasis in the lamina of the visual system of the house fly. Structural homeostasis was also revealed in the context of olfaction in *Drosophila*. Exposure of adult fruit-flies to a high concentration of an odorant for 4 days resulted in behavioral adaptation to the odorant, normal olfactory transduction, and a reduction in olfactory glomeruli size in the antennal lobes (Devaud et al., 2001).

A subcellular evidence of structural homeostasis in the *Drosophila* brain was found in the mushroom body calyx and photoreceptor terminals in the lamina (Kremer et al., 2010; Sugie et al., 2015). Inhibiting action potential firing in antennal lobe projection neurons that connect to mushroom body Kenyon cells increased the size of microglomeruli in the mushroom body calyx (Kremer et al., 2010). Pre-synaptic active zone density also increased, which means that, upon a reduction in neuronal activity, the neurons increase in synapse number to restore function (Kremer et al., 2010). This is evidence of compensatory plasticity. On the other hand, exposing flies to constant light for the first 1–3 days after eclosion decreased the number of synaptic active zones and T-bars from photoreceptors in the lamina (Sugie et al., 2015). Thus, neuronal stimulation induced the disassembly of presynaptic active zones.

The adult fly brain altogether manifests activity-dependent structural homeostasis, as neuronal activity decreased the olfactory glomeruli size and caused synapse elimination, and compensatory plasticity, as the microglomeruli size and synapse number increased in the absence of stimulation (Figure 1).

### Circadian Plasticity and Sleep

Structural changes in the brain have long been known to occur with circadian rhythms. Neurotransmitters were first shown to affect cell size with circadian periodicity (Pyza and Meinertzhagen, 1996). The size of the nuclei of lamina L2 neurons changes with the circadian rhythm, larger in the morning and smaller at night (Gorska-Andrzejak et al., 2005). L2 neuron dendrites are also largest in the morning and smallest in the middle of the night (Weber et al., 2009). However, whether this reflects changes directly controlled by the circadian clock or changes in response to the environment is less clear. For instance, lamina neuron dendritic changes were eliminated when flies were kept in constant light (Weber et al., 2009). Axonal arborizations of clock neurons, such as pigment-dispensing factor (PDF) neurons, are also more complex during the day than at night (Herrero et al., 2020), but activating PDF neurons with TrpA1 increased axonal complexity in the night to the same level as in the morning (Herrero et al., 2020). Thus, changes induced in daytime, by light, could reflect activity-dependent plasticity in clock neurons as well as other neuron types.

Sleep is linked to the circadian clock, and fruit-flies also sleep during night-time. The synapse number and size increase in the brain during waking time, synapse accumulation during the day drives sleep need, and synapse number decreases with sleep (Bushey et al., 2011; Huang et al., 2020). Thus, sleep is required for the homeostatic re-normalization in synapse number (Bushey et al., 2011; Huang et al., 2020). Whether sleep or the lack of it can have further structural consequences in circuits is an interesting question.

### Cell Number Plasticity in the Adult Brain

Cell number plasticity reflects changes in cell proliferation, survival, or death. Apoptosis in the adult fruit-fly brain is limited to the critical period. Dying cells were detected in multiple brain areas, but by the fifth day of adult life, apoptosis was no longer detected (Ito and Hotta, 1992; Kato et al., 2009). In the intact, normal, young brain, glial proliferation was observed adjacently to apoptotic cells, suggesting that apoptosis in the adult brain induces compensatory proliferation in the nearby glia (Kato et al., 2009).

Evidence of adult neurogenesis in adult *Drosophila* was recently reviewed in Li and Hidalgo (2020) and will not be dealt with in detail here. In brief, [^3^H]-thymidine and BrdU experiments labeling cells going through S-phase had suggested that cell proliferation might take place (Technau, 1984, see also Li and Hidalgo, 2020), but this could reflect polyploidy instead (Nandakumar et al., 2020). Importantly, the developmental neuroblasts that make the adult brain are eliminated through either apoptosis or cell cycle exit in the larva or pupa, before the adult ecloses (Siegrist et al., 2010; Li and Hidalgo, 2020). Thus, in the absence of neuroblasts, it was unclear how neurogenesis could proceed. A careful temporal profile of BrdU pulses applied to the adult brain showed that not only there are cells in S-phase in the adult brain but also the number of labeled cells critically increased over time (Kato et al., 2009). This was robust evidence of cell division in the adult brain. Cell proliferation was also reported using clonal analyses (Kato et al., 2009; Fernandez-Hernandez et al., 2013; Foo et al., 2017) and cell cycle markers (Li et al., 2020). The incidence of cell proliferation increased with genetic manipulations and injury (Kato et al., 2009; Fernandez-Hernandez et al., 2013). Injury in the optic lobe or central brain by stabbing with a needle and antennal ablation induced the proliferation of glial cells (Kato et al., 2009; Fernandez-Hernandez et al., 2013). Injury-induced cell proliferation seemed to be restricted to a critical period in the first 10 days of young flies, although this remains to be confirmed. The dividing cells were shown to be, in some cases, glia but could also include progenitor cells, as in the normal, intact, adult brain, there are cells that express the common neural stem cell marker *deadpan* (Fernandez-Hernandez et al., 2013; Li et al., 2020).

These findings altogether mean that cell number plasticity in the adult *Drosophila* brain includes both loss and generation of new cells (Figure 1). There is suggestive evidence for a critical period in cell number plasticity in the young fly, but this remains to be confirmed.

## Structural Brain Plasticity Is Common in Insects

Structural brain plasticity occurs in other insects and could be widespread. In moths, pre-exposure to bat sound increased the volume of some glomeruli in antennal lobes and mushroom body calyces (Anton et al., 2016). In bumblebees kept in complete darkness for 7 days, lack of visual stimulation increased the relative volume of antennal lobes, mushroom body calyces, and neuropils (Jones et al., 2013). In honeybees, the synaptic bouton number in the calyx of the lip—one of the mushroom body compartments—increased when the bees were reared in impoverished conditions with reduced social interactions and olfactory and visual stimuli compared to bees reared in the hive (Cabirol et al., 2017). The structure of both the lip and dense collar (also mushroom body compartments) were also affected by foraging behavior. The volume and bouton number of the lip and dense collar correlated positively with the time spent foraging (Cabirol et al., 2018). These and further findings (Hourcade et al., 2009) showed that, in honeybees, the volume and synapse number of both antennal lobes and mushroom bodies can be modified by environmental stimuli, which, in turn, modified the behavior.

Thus, like in the mammalian brain, environmental stimuli induce structural brain plasticity in multiple insect species, including fruit-flies, bumblebees, honeybees, and moths. This suggests that structural brain plasticity is a fundamental property of how brains work and are modified through experience. In both insects and humans, the brain modules involved in olfaction and learning and memory were found to be plastic. Remarkably, contrary to the limited neurogenic sites of the human brain but similar to its cortical brain plasticity, plasticity appears to be widespread throughout the insect brain.

## Molecular Mechanisms of Structural Brain Plasticity

The molecular mechanisms underlying structural brain plasticity are beginning to be uncovered (Table 1 and Figure 1). Pioneering work first addressed this question by testing genes involved in phototransduction and learning and memory. Mutations in genes required for phototransduction such as *norpA* (causing loss of phospholipase-C) and *hdc^*jk*910^* (deficient in histamine, the main neurotransmitter in the retina) obliterated the differences in brain volume observed when rearing fruit-flies in constant light vs. darkness (Barth and Heisenberg, 1997). This meant that visual experience is required for light-induced structural brain plasticity in the optic lobes. By contrast, mutations in genes involved in cAMP signaling *dunce* (*dnc*), *rutabaga* (*rut*), and *amnesiac* (*amn*), involved in learning, did not cause volume changes in the optic lobe with light rearing, but *dnc* and *amn* did cause volume changes in the mushroom body calyx (Barth and Heisenberg, 1997). Furthermore, when kept in the dark, wild-type flies in large social groups had larger calyces than flies housed in smaller groups, but this effect was lost in all three mutants (Barth and Heisenberg, 1997). It was recently shown that rut, which encodes Ca^2+^/CaM-dependent adenyl cyclase, is also required for structural plasticity at the calyx. In fact, long-term memory modifies the size and number of microglomeruli in the calyx, but this effect was abolished in *rut* mutants or by blocking protein synthesis (Baltruschat et al., 2021). Thus, cAMP regulates plasticity in calyx but not the visual system, which means that distinct molecular pathways may underlie structural plasticity in distinct brain regions (Barth and Heisenberg, 1997).

**TABLE 1:** Genes involved in structural brain plasticity in *Drosophila*.

	**Gene**	**What does it encode?**	**What does it do in structural brain plasticity?**	**References**
Classical pathways Involved in neuro-transmission or learning	norpA	Phospholipase C Regulates phototransduction	Regulate volume of lamina.	Barth and Heisenberg, 1997
	hdc	Histidine decarboxylase catalyses the decarboxylation of histidine to form histamine that is involved in neurotransmission	Regulate volume of lobula plate.	Barth and Heisenberg, 1997
	Dunce (dnc)	cAMP phosphodiesterase	Regulates volume of the mushroom body calyx.	Barth and Heisenberg, 1997
	Rutabaga (rut)	Homolog of adenylate cyclase generates cAMP	Regulates volume of MB calyx and synapse formation during learning.	Barth and Heisenberg, 1997; Baltruschat et al., 2021
	Amnesiac (amn)	Suppressor of dunce	Regulates the volume of the MB calyx	Barth and Heisenberg, 1997
Neuro- and glio- genesis	Eiger	Type II transmembrane protein, homolog of tumo r necrosis factor (TNF).	Required for cell proliferation in response to stubbing injury in adult brain.	Kato et al., 2009
	dMyc	homolog of vertebrate MYC, a transcription factor	Promote proliferation of Dpn^+^ cells.	Fernandez-Hernandez et al., 2013
	miR-31a	microRNA-31a	Induces cell apoptosis of glia in young adult flies.	Foo et al., 2017
Circadian	Pigment dispersing factor (PDF)	Neuropeptide	Transmits circadian information from ventrolateral neurons (LNvs).	Herrero et al., 2020
	Bruchpilot (Brp)	Active zone scaffold protein	Promotes synapse formation and sleep need	Huang et al., 2020
Toll signaling	Toll-2 (also known as 18 wheeler)	Toll-2 of the family of *Drosophila* Toll receptors (Toll-1 to Toll-9)	Regulates cell survival, and proliferation. Toll-2 loss causes axon and dendrite degeneration.	Li et al., 2020.
	MyD88	Canonical downstream adaptor of Tolls	Promotes cell survival and keep progenitors quiescent	Li et al., 2020
	Weckle (Wek)	Downstream adaptor of Tolls	Promotes cell death and cell proliferation	Li et al., 2020
	Yorkie (Yki)	Transcriptional coactivator, ortholog of mammalian yes-associated protein (YAP)	Promotes cell proliferation	Li et al., 2020

Several genes promoting cell proliferation in the adult *Drosophila* brain were identified. The TNF homolog Eiger is required for cell proliferation in response to injury in the adult brain, as cell proliferation was not induced in injured *eiger* mutants (Kato et al., 2009). Over-expression of the oncogene *dMyc* in the adult brain increased the proliferation of Dpn^+^ progenitor cells (Fernandez-Hernandez et al., 2013). Mutant microRNA miR-31a induced the apoptosis of glia in young adult flies, which was followed by compensatory proliferation (Foo et al., 2017). So, these data altogether suggested that, in the adult brain, common fundamental cellular processes can also drive cell number adjustments, at least upon cell death or injury.

In clock neurons, the expression of PDF increases during the day, positively correlating with an increase in axonal complexity of PDF neurons (Herrero et al., 2020). Conversely, conditional PDF knock-down in the adult prevents structural plasticity. These findings altogether demonstrate that PDF regulates structural plasticity in clock neurons (Herrero et al., 2020).

*Drosophila* neurotrophins are encoded by the *spätzle* (*spz*) paralog gene family, which includes *Drosophila* neurotrophin-1 (DNT1, also called spz-2), DNT2/spz-5, and Spz-1 (Zhu et al., 2008; Sutcliffe et al., 2013; Foldi et al., 2017). During nervous system development, DNTs promote neuronal survival, cell death, connectivity, structural synaptic plasticity, and compensatory plasticity (Zhu et al., 2008; Sutcliffe et al., 2013; Foldi et al., 2017; Ulian-Benitez et al., 2017). Mammalian neurotrophins bind Trk and p75 receptors, but DNTs bind kinase-free Trk homolog encoded by the *kekkon* genes and Toll receptors (Mandai et al., 2009; Ulian-Benitez et al., 2017). Toll-1 in *Drosophila* is responsible for dorso-ventral body axis in embryogenesis and for innate immunity (Hashimoto et al., 1988; Lemaitre et al., 1996), and it led to the discovery of TLRs throughout most organisms. Tolls and TLRs not only have universal, evolutionarily conserved functions controlling innate immunity, but they also have non-immune functions (Anthoney et al., 2018). There are nine Tolls in the *Drosophila* genome, and during embryonic and larval nervous system development, at least some Tolls have been shown to be involved in connectivity, cell number plasticity, and structural synaptic plasticity (Zhu et al., 2008; McIlroy et al., 2013; Ballard et al., 2014; Ward et al., 2015; McLaughlin et al., 2016; Foldi et al., 2017; Ulian-Benitez et al., 2017; Li et al., 2020). At least seven Tolls are expressed in the adult brain (Toll-1, −2, −3, −5, −6, −7, and −8), in overlapping but distinct expression patterns that coincide with brain anatomical domains (Li et al., 2020; Figure 1).

**FIGURE 2:**
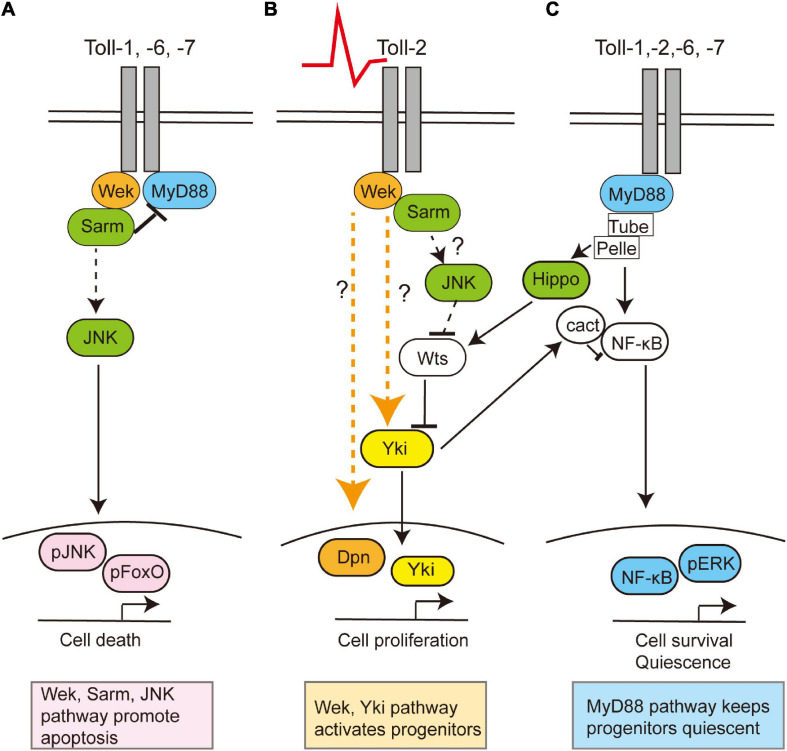
Toll receptors can regulate cell survival, death, quiescence, or proliferation *via* alternative signaling pathways. Toll receptors share common functions, but they can also each elicit distinct cellular outcomes. This is a summary of data evidence on signaling by Toll-1, −2, −6, and −7 in the central nervous system in embryonic, larval, and/or pupal development and Toll-2 in the adult brain. **(A)** During development, Toll receptors can promote cell apoptosis *via* Wek, Sarm, and JNK, **(B)** but in the adult brain, Toll-2 receptors can also function *via* Wek and Yki to promote cell proliferation. Question marks denote how or whether Wek signals remain unknown. **(C)** Toll receptors can promote cell survival *via* MyD88-NFkB signaling both throughout development and in the adult. Moreover, adult progenitor cells are normally kept quiescent *via* MyD88. In other contexts, the activation of Toll by gram-positive bacteria prevents proliferation by activating the Hippo pathway, thus inhibiting Yki, although whether this is also the case in the brain is unknown.

Tolls can promote either cell quiescence, proliferation, neuronal survival, or death, depending on time, cell type, and cell context, in the CNS (McIlroy et al., 2013; Foldi et al., 2017; Li et al., 2020; Figure 2). Canonical Toll signaling proceeds *via* the MyD88 adaptor, leading to the activation of NF-κB homologs Dorsal and Dif downstream, which in the CNS promote cell survival (Foldi et al., 2017; Anthoney et al., 2018). Tolls can also function *via* Weckle (Wek) and Sarm to promote cell death (Foldi et al., 2017). Sarm is an evolutionarily conserved inhibitor of MyD88, and it induces apoptosis *via* JNK downstream (Foldi et al., 2017). Tolls bind Wek, which binds Sarm facilitating its inhibition of MyD88 (Foldi et al., 2017). Sarm also has NAD-ase activity which drives axonal degeneration (Carty and Bowie, 2019). Toll-2 also regulates cell proliferation during development, *via* Wek and Yorkie (Yki) (Li et al., 2020). Concerted knock-down of multiple *Tolls* throughout development causes dramatic reductions in brain size, most likely due to combined increased cell death and reduced cell proliferation (Li et al., 2020). Although Tolls can have redundant functions, they are not equal, and they can lead to distinct cellular outcomes. For example, in the pupal CNS, Toll-1 and Toll-6 can induce both cell survival and cell death, but Toll-1 has a stronger pro-apoptotic effect, whereas Toll-2 does not induce cell death (Foldi et al., 2017; Li et al., 2020).

Toll-2, at least, is responsible for generative processes underlying structural brain plasticity in the adult (Li et al., 2020). It is neuroprotective, and *Toll-2* knock-down causes axonal misrouting, loss of axons and dendrites, and loss of neurons and impairs behavior (Li et al., 2020). Toll-2 promotes neurogenesis in the adult brain, as the conditional over-expression of *Toll-2* restricted to the adult promotes cell proliferation v*ia* Yki, and increases cell number and brain size (Li et al., 2020). Neuronal activation with TrpA1 also increases cell number, and this depends on Toll-2 (42). There are Dpn^+^ progenitors in the adult *Drosophila* brain (Li et al., 2020), which express MyD88. Toll signaling *via* MyD88 inhibits the proliferation of progenitor cells, whereas signaling *via wek* promotes cell proliferation (Li et al., 2020). Similarly, in immunity, Toll signaling *via* MyD88 activates Hippo signaling, inhibiting Yki and cell proliferation (Liu et al., 2016). Thus, Toll signaling *via* MyD88 keeps progenitor cells quiescent, and neuronal activity or Toll-2 signaling can activate an alternative Wek downstream pathway that promotes cell proliferation *via* Yki (Li et al., 2020; Figure 2). Thus, depending on the availability of downstream adaptors, Toll receptors can function *via* alternative signaling pathways, with distinct cellular outcomes (Foldi et al., 2017). What enables Tolls to switch between signaling *via* MyD88, Sarm, or Wek to promote cell survival and quiescence, apoptosis, or cell proliferation, respectively, is not known.

The topographic expression of Tolls together with the multiple possible outcomes downstream (Figures 1, 2) contributes to the formation of the distinct brain modules in development and could have driven the formation and diversification of distinct brain types in the course of evolution. Importantly, it enables them to regulate structural plasticity independently in different brain domains. In fact, the cell number in the optic lobe medulla and central brain was found to be more plastic than mushroom body Kenyon cells (Li et al., 2020). By regulating the cell number independently in distinct brain domains (e.g., visual, olfactory), Tolls can match a sensory experience (e.g., vision and olfaction) to a structural change. In contrast to the limited neurogenic niches of the mammalian brain, cell cycling markers and changes in cell number were found spread out through the *Drosophila* brain, reminiscent of widespread adult neurogenesis in zebrafish. These findings meant that the cell number in the adult fruit-fly brain is plastic, and experience can elicit structural changes in the brain *via* Toll receptor signaling.

Sleep need can be driven by increasing the levels of the active zone scaffold protein Bruchpilot (Brp) (Huang et al., 2020). Increasing the levels of Brp led to a dose-dependent increase in synapse formation, and this was sufficient to quantitatively tune sleep patterns reminiscent of sleep deprivation (Huang et al., 2020). When knocking down sleep-regulating genes, such as *wide awake*, *insomniac*, or *fumin*, the expression level of the active zone scaffold protein Bruchpilot significantly increased in a brain-wide manner (Huang et al., 2020). Moreover, knocking down brp expression in R2 neurons in flies that otherwise over-expressed Brp was sufficient to partially rescue a sleepless behavior and a deficient short-term memory, thus demonstrating the importance of these neurons and Brp in the regulation of sleep (Huang et al., 2020).

Toll and Spz are also involved in regulating sleep homeostasis. Astroglial calcium increases with sleep need and is required for sleep rebound after sleep deprivation (Blum et al., 2021). It has been proposed that astrocytes secrete Spz, which signals *via* Toll-1 in R5 neurons to promote a sleep rebound (Blum et al., 2021). The intracellular Ca^2+^ level in R5 neurons significantly increased after sleep deprivation, and knocking down Toll suppressed this increase (Blum et al., 2021). Moreover, knocking down *Spz* in astroglia or *Toll* in R5 neurons reduced the homeostatic sleep rebound (Blum et al., 2021). However, whether Spz and Toll-1 can also cause structural modifications in R5 neurons and whether this is linked to sleep homeostasis was not investigated.

To conclude, evidence indicates that these and other yet-to-be-discovered molecular mechanisms underlie structural changes in the fly brain, modifying neural circuits in response to experience to deliver appropriate behavior, learning, and sleep and to adapt to the environment.

## Similarities Between Tolls and Mammalian TLRs in Structural Brain Plasticity

There are 10 TLRs in the human brain. Investigation of TLR function in the mammalian brain has mostly focused on their immune-like functions in microglia and astrocytes (Okun et al., 2009, #76; Okun et al., 2011, #87; Shmueli et al., 2018, #308; Anthoney et al., 2018, #35; Chen et al., 2019, #312; Donnelly et al., 2020, #311), but TLRs are also expressed in neural stem cells, NG2 glia, and neurons throughout development and in the adult. TLRs also have non-immune functions in the mammalian nervous system (Rolls et al., 2007, #78; Okun et al., 2010, #221; Chen et al., 2019, #312; Donnelly et al., 2020, #311). They are required to either promote or prevent neural stem cell proliferation and differentiation into neurons or glia and to regulate cell survival or death, neurite growth or retraction, synaptogenesis, and the compensation of spine density and size. In neurons, they can regulate slow cellular processes through the canonical MyD88 and ERK signaling pathways and gene expression (Rolls et al., 2007; Okun et al., 2010) and also fast neuronal action (e.g., < 1 min) in interaction with channels (e.g., TrpA1 and TrpV1), enabling fast responses to sensory stimuli, such as heat, pain, and itch (Donnelly et al., 2020, #311). This repertoire of functions reveals that TLRs could be involved in regulating brain function and plasticity independently of immunity.

*Drosophila* Tolls can carry out overlapping yet distinct functions (Foldi et al., 2017). Similarly, in mammals, TLR-2, −3, and -4 regulate neurogenesis and cell differentiation; TLR-7, −8, and -9 can induce apoptosis; and TLR-3, −7, and -8 regulate arborizations through neurite growth and retraction, spine density, and size (Ma et al., 2006; Cameron et al., 2007; Rolls et al., 2007; Okun et al., 2010; Chen et al., 2019). Thus, not all Tolls and TLRs are equal, and they may each elicit distinct cellular outcomes.

Whether *TLRs* are expressed topographically in the brain, like *Drosophila* Tolls, is not yet known. There is evidence that *TLRs* are expressed with distinct temporal profiles from brain development to the adult brain (Ma et al., 2006; Kaul et al., 2012; Barak et al., 2014; Arnaboldi et al., 2020). They might be expressed in partly overlapping and at least partly distinct cell types in the brain, as their loss of function causes region-specific behavioral phenotypes. For instance, TLR4 regulates the levels of neuromodulators in the frontal cortex and hippocampus (Femenia et al., 2018). Mice with altered TLR-3 or -4 function have altered spatial navigation, learning and memory, anxiety, and social interactions (Okun et al., 2012). However, these observations were made in mutant mice, and it is compelling to find out whether conditional alterations in TLR function restricted to the adult would also influence behavior and in a spatially dependent manner. Finding out whether TLRs are distributed topographically like in *Drosophila* would explain the experience-dependent regulation of brain plasticity and the consequences in behavior.

The endogenous ligands that could regulate non-immune neuronal TLR functions in the sterile, undamaged brain are unknown. Neurotrophins are ligands for Tolls in *Drosophila*, but whether they can also bind mammalian TLRs remains unexplored. Interestingly, *TLR4* knock-out mice with anxiety behavior also had an altered expression of BDNF in the frontal cortex and hippocampus (Femenia et al., 2018). Intriguingly, in cell culture, human neurotrophins BDNF and NGF can modify human TLR signaling in response to their endogenous ligands and induce TLR-4 signaling in the absence of any other ligand (McIlroy et al., 2013; Foldi et al., 2017; Li et al., 2020). This means either that binding of NGF and BDNF to Trk or p75 receptors modifies TLR signaling or that NGF and BDNF can directly bind at least TLR4 (Foldi et al., 2017). It is compelling to find out whether TLRs could also respond to NGF or BDNF *in vivo* in the mammalian brain.

Understanding TLR function in the brain is important, as alterations result in brain diseases, including anxiety, neuropsychiatric disorders, schizophrenia, autism, Alzheimer’s disease, autoimmune diseases (e.g., multiple sclerosis), and stroke (Okun et al., 2011).

## Conclusion

There is abundant evidence of structural brain plasticity in the *Drosophila* brain, involving modifications to regional volumes, cell number, cell shape, and synapses, altogether modifying neural circuits. In *Drosophila*, Toll receptors regulate structural brain plasticity topographically, activating alternative signaling pathways downstream that can promote cell proliferation, quiescence, survival, or death. In this way, Tolls enable the link between sensory experience and structural brain change. There are striking similarities in the way *Drosophila* Tolls and mammalian TLRs function in the brain, including through distinct Toll-specific functions, cellular outcomes, and consequences in behavior. Although mammalian TLRs have been investigated mostly in the context of immunity, they also have non-immune functions in the sterile, undamaged brain, and these functions could be crucial to understanding brain diseases. Communication between *Drosophila* and mammalian findings can expedite the understanding of structural change in the human brain—in health and disease.

## Author Contributions

GL and AH wrote and revised the manuscript. Both authors contributed to the article and approved the submitted version.

## Conflict of Interest

The authors declare that the research was conducted in the absence of any commercial or financial relationships that could be construed as a potential conflict of interest.
